# Potential Role for MATER in Cytoplasmic Lattice Formation in Murine Oocytes

**DOI:** 10.1371/journal.pone.0012587

**Published:** 2010-09-07

**Authors:** Boram Kim, Rui Kan, Lynne Anguish, Lawrence M. Nelson, Scott A. Coonrod

**Affiliations:** 1 Baker Institute for Animal Health, College of Veterinary Medicine, Cornell University, Ithaca, New York, United States of America; 2 Intramural Research Program on Reproductive and Adult Endocrinology, National Institute of Child Health and Human Development, Bethesda, Maryland, United States of America; McGill University, Canada

## Abstract

**Background:**

*Mater* and *Padi6* are maternal effect genes that are first expressed during oocyte growth and are required for embryonic development beyond the two-cell stage in the mouse. We have recently found that PADI6 localizes to, and is required for the formation of, abundant fibrillar Triton X-100 (Triton) insoluble structures termed the oocyte cytoplasmic lattices (CPLs). Given their similar expression profiles and mutant mouse phenotypes, we have been testing the hypothesis that MATER also plays a role in CPL formation and/or function.

**Methodology/Findings:**

Herein, we show that PADI6 and MATER co-localize throughout the oocyte cytoplasm following Triton extraction, suggesting that MATER co-localizes with PADI6 at the CPLs. Additionally, the solubility of PADI6 was dramatically increased in *Mater^tm/tm^* oocytes following Triton extraction, suggesting that MATER is involved in CPL nucleation. This prediction is supported by transmission electron microscopic analysis of *Mater^+/+^* and *Mater^tm/tm^* germinal vesicle stage oocytes which illustrated that volume fraction of CPLs was reduced by 90% in *Mater^tm/tm^* oocytes compared to *Mater^+/+^* oocytes.

**Conclusions:**

Taken together, these results suggest that, similar to PADI6, MATER is also required for CPL formation. Given that PADI6 and MATER are essential for female fertility, these results not only strengthen the hypothesis that the lattices play a critical role in mediating events during the oocyte-to-embryo transition but also increase our understanding of the molecular nature of the CPLs.

## Introduction

A unique feature of mammalian oocytes is that transcription ceases upon oocyte maturation [1] and does not resume until embryonic transcription becomes activated in the early embryo [2]–[5]. During this period of transcriptional quiescence, the oocyte must rely on maternal factors, structures, and organelles that have accumulated in the oocyte during growth to mediate this critical period, often called the oocyte-to-embryo transition (OET). In non-mammalian species, mutation analysis has identified a large number of factors, called maternal effect genes (MEGs), which are synthesized and accumulate in the oocyte and then persist in the early embryo where they are required for embryonic development [6], [7]. Phenotypic analysis of mouse knockout models has recently lead to the identification of several mammalian MEGs such as Maternal Antigen That Embryos Require (MATER) and Peptidylarginine Deiminase 6 (PADI6), two highly-abundant oocyte-restricted proteins that are essential for embryonic development beyond the two-cell stage [8], [9].

MATER (gene name, NLRP5) was originally identified as an antigen that is involved in a mouse autoimmune oophoritis [10]. Recently, MATER (mother in Latin) has been identified as a component of the SCMC (subcortical maternal complex) along with other maternal factors including FILIA (daughter in Latin), FLOPED, and TLE6 [11]. Additionally, PADI6 has also been putatively identified as a component of the SCMC complex. While FILIA is thought to play a role in chromosome stability during embryogenesis [12], the role of MATER remains to be elucidated.

PADI6 was originally cloned from the mouse oocyte proteome due to its abundance in metaphase II-arrested oocytes and its oocyte-restricted expression pattern [9]. Interestingly, PADI6 is localized to, and required for, the formation of an abundant, oocyte- and early embryo-restricted structure, the cytoplasmic lattices (CPLs or lattices) [9], [13]. The lattices are composed of 5–7 parallel fibers with each fiber containing a repeating unit of ∼20 nm [14]. The bundled fibers are first observed at early stages of oocyte growth (30–40 µm) [15] and persist in the early embryo until the blastocyst stage [16]. CPLs were found to be resistant to Triton-X-100 (Triton), thus, extraction with this detergent provides a valuable tool for studying CPL associated proteins [14], [17]. While CPLs have been observed by electron microscopy since the 1960s, their function remains poorly understood. Based on electron microscopy and biochemical analysis, a number of older reports predicted that the lattices may function as yolk granules [18] or as a ribosomal storage site [15], [19]–[23], with the latter hypothesis being supported by recent data from our lab [24].

Interestingly, *Padi6* and *Mater* share many similar properties. For example, the expression of both maternal genes is regulated by the basic helix-loop-helix transcription factor, FIGLA (Factor in the germline alpha) [25] and is restricted to oocytes and early embryos in mouse. Microarray analysis [26] along with previous studies [9], [27], [28] suggest that both transcripts appear in the oocyte at the primordial/primary follicle stage and then abruptly disappear around meiotic maturation. MATER and PADI6 protein expression roughly parallels that of their transcripts in oocytes; however, protein levels persist at high levels throughout preimplantation development until the blastocyst stage [9], [27]. Additionally, analysis of *Padi6^−/−^* and *Mater^tm/tm^* (tm: targeted mutation) female mice indicates that the phenotypes of embryos conceived from these two mutants are strikingly similar with a developmental arrest occurring at the two-cell stage; likely due to abnormal embryonic genome activation (EGA) as demonstrated by reduced levels of BrUTP and TRC transcripts in these embryos [8], [13], [24].

Based on these similarities, we hypothesized that, similar to PADI6, MATER may also play a role in CPL formation. Here we show that PADI6 and MATER co-localize throughout the oocyte cytoplasm following Triton extraction and appear to both be associated with large complexes of similar molecular weight. Additionally, the solubility of PADI6 (a CPL marker) is greatly increased in *Mater* hypomorph oocytes, suggesting that lattices are significantly reduced in *Mater^tm/tm^* oocytes. As a more direct confirmation of the requirement of MATER for CPL formation, we show by electron microscopy that the volume of CPLs is reduced 9.65-fold in germinal vesicle (GV) stage *Mater^tm/tm^* oocytes. Taken together, these results suggest that MATER is required for CPL formation. Studies are currently underway to test the hypothesis that MATER's role in CPL synthesis is due to direct interactions with other CPL-associated factors and to further investigate CPL function using the *Mater^tm/tm^* mouse oocyte model.

## Results

### PADI6 and MATER co-localize in oocytes and early embryos and appear to associate with high molecular weight complexes

**Figure 1:**
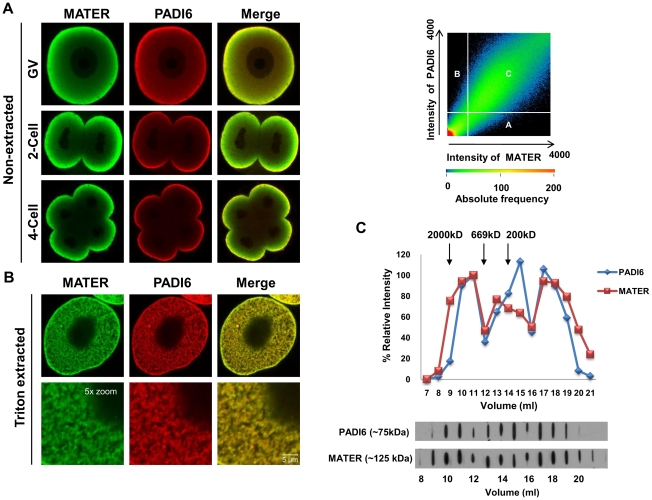
Co-localization of MATER and PADI6 in non-extracted and Triton X-100 extracted GV oocytes and early embryos. (**A**) Confocal microscopic images show non-extracted GV-stage oocytes, 2-cell, and 4-cell embryos after fixation, permeabilization and incubation with anti-MATER (green) and anti-PADI6 (red) antibodies. (**B**) GV oocytes extracted with 0.1% Triton X-100 and stained with MATER (green) and PADI6 (red) antibodies. Merged images highlight MATER and PADI6 co-localization. Scatter plot indicates a degree of co-localization of MATER and PADI6. Region A shows pixels with high MATER (green) intensities, region B shows pixels with high PADI6 (red) intensities, and region C shows pixels with both high MATER (green) and PADI6 (red) intensities. Mander's overlap coefficient: 0.97, Pearson's correlation coefficient: 0.7. (**C**) CD1 mouse oocyte lysates were chromatographed by FPLC. Eluted fractions (1 ml) were analysed by immunoblotting with antibodies to PADI6 and MATER. Densitometry was used to generate a graph and the values in fraction 11 were set at a relative intensity of 100%. Elution of each protein standard is indicated by arrow.

The localization of MATER and PADI6 has been reported previously [9], [27], [28]. Given that PADI6 primarily localizes to the CPLs [9] and is required for CPL formation [13], we believe that PADI6 represents a good marker for the CPLs. Therefore, to begin testing the hypothesis that MATER plays a role in CPL function, we first carried out confocal immunofluorescence microscopy (CIM) to determine if these two maternal factors co-localize. Oocytes and embryos were fixed with paraformaldehyde (PFA), permeabilized and stained with anti-PADI6 and anti-MATER antibodies. The results showed that MATER and PADI6 appear to strongly co-localize in the cortex and, to a lesser degree, in the cytoplasm of GV stage oocytes. Co-localization was also observed at the non-opposed cortex regions of 2-cell and 4-cell stage embryos with less co-localization being observed throughout the cytoplasm in the early embryos (Fig. 1A). Interestingly, in two and four cell embryos, PADI6 staining is more strongly localized to the cortex than MATER, which can be seen to penetrate deeper into the cytoplasm. CPLs are Triton insoluble structures. Therefore, to more directly test the hypothesis that MATER localizes to the CPLs, we next extracted GV oocytes with 0.1% Triton for 10 min and then carried out co-localization analysis using CIM. Following Triton extraction, the cytoplasmic signal of MATER and PADI6 was greatly enhanced throughout the oocyte cytoplasm, thus providing an indirect line of evidence that MATER is associated with the CPLs (Fig. 1B). To test if MATER and PADI6 co-localize in the extracted oocytes, co-localization analysis was performed with Zeiss 2007 software and the scatterplot shows the degree of co-localization between MATER and PADI6 in the extracted oocytes. Mander's overlap and Pearson's correlation coefficients (0.97 and 0.7, respectively) confirm the high degree of colocalization between MATER and PADI6 (Fig. 1B).

To further strengthen the potential association between MATER and PADI6, we next resolved proteins from GV stage outbred CD1 mouse oocyte extracts using fast protein liquid chromatography (FPLC) and eluted fractions were analyzed by immunoblot using MATER and PADI6 antibodies. Results show that PADI6 and MATER appear to co-elute in three different fractions. An initial high molecular weight fraction is seen between 2000 kD and 669 kD, a second fraction is observed between 669 and 220 kD, and a third peak is also seen that is below the 200 kD molecular weight marker. Additionally, a fraction of PADI6 also appears to elute independent from MATER just below the 200 kD marker. The observation that PADI6 and MATER appear to co-elute in high (∼1,000 to 2,000) molecular weight fractions supports the hypothesis that these two maternal proteins are associated with a supramolecular complex (Fig. 1C).

### The Triton X-100 solubility of PADI6 is increased in *Mater^tm/tm^* oocytes

**Figure 2:**
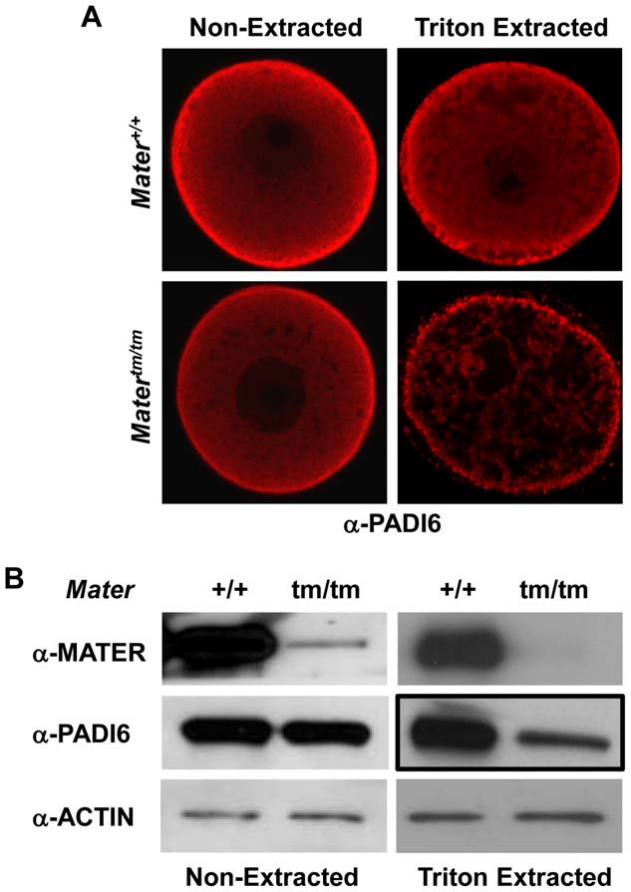
PADI6 Triton X-100 solubility is increased in *Mater^tm/tm^* GV-stage oocytes. (**A**) Confocal analysis shows *Mater^+/+^* and *Mater^tm/tm^* GV-stage oocytes prior to, and following, extraction with 0.1% Triton X-100. Oocytes were incubated with PADI6 antibodies (red). (**B**) Western blotting shows expression of MATER and PADI6 protein in *Mater^+/+^* and *Mater^tm/tm^* GV-stage oocytes prior to, and following, 0.1% Triton X-100 extraction. Isolated oocytes were either extracted or not extracted with Triton, and then evaluated by Western blotting using either anti-MATER, anti-PADI6, or anti-β actin antibodies.

The oocyte CPLs cannot be visualized by EM in *Padi6* null mouse oocytes [24], suggesting that the CPLs do not form in the absence of PADI6 and that proteins which normally associate with the CPLs are rendered more soluble in the mutant eggs. This idea is supported by our previous finding that the Triton solubility of ribosomal components, such as S6, is greatly enhanced in *Padi6*-null eggs [24]. Therefore, we decided to use a similar type of approach in *Mater* mutant oocytes to more directly test the hypothesis that MATER plays a role in CPL formation and function. We predicted that if CPLs do not form in *Mater^tm/tm^* oocytes, then PADI6 (a CPL-associated protein) should become more soluble following Triton extraction. GV oocytes were either not extracted or extracted with 0.1% Triton for 10 minutes and prepared for CIM as described above. Results showed that, in non-extracted oocytes, the level and localization of PADI6 was similar between *Mater^+/+^* and *Mater^tm/tm^* oocytes with slightly reduced PADI6 level at the cortex in *Mater^tm/tm^* oocytes. However, PADI6 levels were dramatically reduced in *Mater^tm/tm^* oocytes following Triton extraction (Fig. 2A). In order to better quantitate these findings, we carried out western blot analysis of non-extracted and Triton extracted oocytes. In line with our confocal findings, immunoblot analysis showed that PADI6 levels were similar between wild type and *Mater* hypomorph oocytes. However, following Triton extraction, analysis of the insoluble fraction found that, while levels of β-actin were similar, image densitometry quantification revealed that levels of PADI6 were reduced by ∼50% in the mutant compared to the control wild type oocytes (Fig. 2B). Taken together, these results suggest that the lattices do not form (or form to a lesser degree) in the absence of MATER, thus releasing PADI6 from the insoluble fraction.

### Morphometric electron microscopic analysis reveals that CPL volume is greatly reduced in M*ater^tm/tm^* oocytes

**Figure 3:**
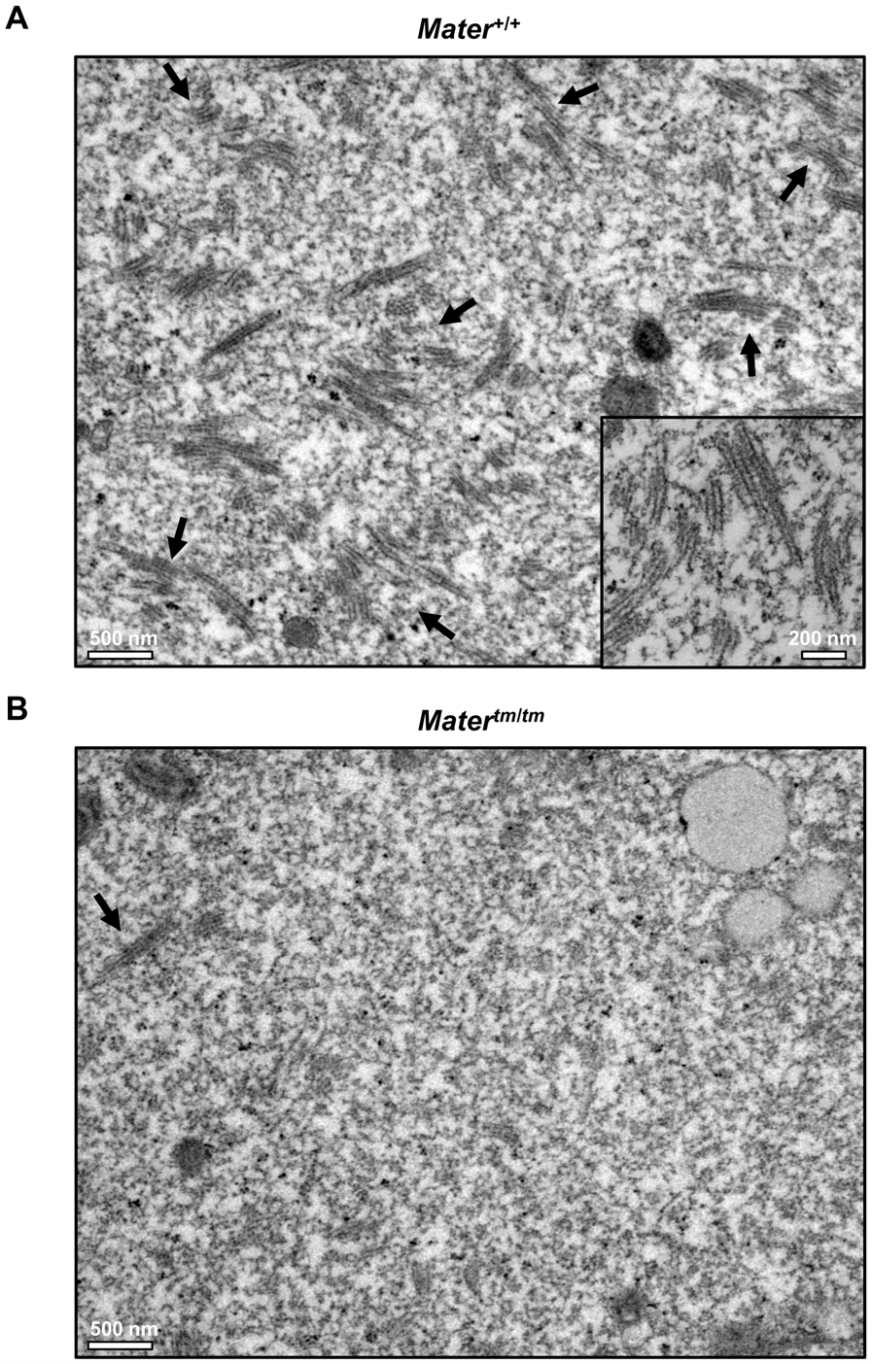
EM analysis reveals that the volume fraction of CPLs is reduced in *Mater^tm/tm^* oocytes. (**A**) Representative TEM image (x11,500) of the ultrastructure of *Mater^+/+^* GV oocytes. A high magnification (x26,500) image of the lattice structure is shown in the inset. (**B**) Representative TEM image (x11,500) showing the ultrastructure of *Mater^tm/tm^* GV oocytes. Arrows in **A** and **B** indicate CPLs.

**Figure 4:**
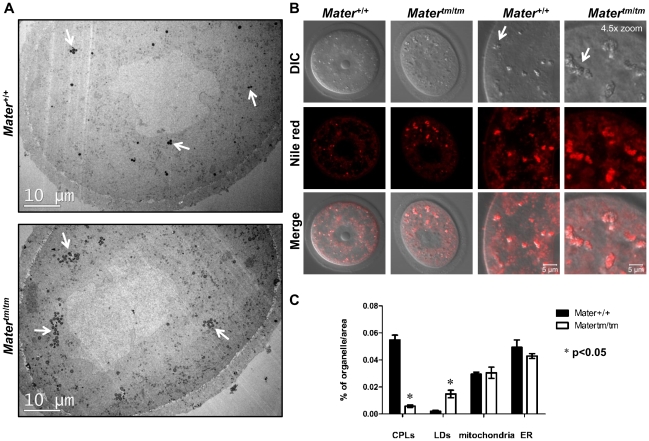
*Mater^tm/tm^* oocytes display elevated levels of lipid droplets. (**A**) Low magnification (x1,700) TEM images of *Mater^+/+^* and *Mater^tm/tm^* GV-stage oocytes. Oocytes were prepared for TEM as above. White arrows point to LDs. (**B**) Confocal images of GV-stage live *Mater^+/+^* and *Mater^tm/tm^* oocytes following Nile red staining. DIC images show morphology of LDs in oocytes. Close up images (Right panel) highlight LD aggregates. Arrows indicate LDs. (**C**) Quantitation of the volume fractions of CPLs, LDs, mitochondria, and ER in *Mater^+/+^* and *Mater^tm/tm^* GV-stage oocytes. Mean ± SEM is indicated. *P* value is <0.004 for CPLs, <0.04 for LD, >0.05 for mitochondria, and ER.

In order to directly test the hypothesis that MATER is required for CPL formation, we next performed morphometric analysis of the CPLs in *Mater^+/+^* and *Mater^tm/tm^* oocytes using transmission electron microscopy (TEM). GV oocytes were collected from three *Mater^+/+^* and three *Mater^tm/tm^* female mice, fixed, embedded with resin, and ultrathin sections of the blocks were placed on a grid. The oocytes were then imaged using TEM and quantitation of the CPLs and other organelles was then carried out using the point-counting method [29]. Results showed that the average volume fraction of CPLs per area was 5.5% for *Mater^+/+^* oocytes and 0.057% for *Mater^tm/tm^* oocytes, thus the volume fraction of CPLs in *Mater^tm/tm^* oocytes was reduced by 9.56 fold when compared to *Mater^+/+^* oocytes. Standard error of mean (SEM) of the CPL volume fraction for *Mater^+/+^* oocytes was 0.0035 and for *Mater^tm/tm^* oocytes was 0.0009 (Fig. 3 and Fig. 4C). This result provides direct support of our hypothesis that MATER is required for CPL formation. It is important to note that a residual amount of MATER protein is present in *Mater^tm/tm^* oocytes (See Fig. 2B and Discussion below), thus possibly explaining why some lattices are observed in hypomorph oocytes.

Interestingly, while scoring CPL volume by TEM, we also noticed that there was an apparent increase in the volume of lipid droplets in *Mater^tm/tm^* oocytes when compared to *Mater^+/+^* oocytes (Fig. 4A). To quantify this observation, we then carried out morphometric analysis of the LDs in the wild type and mutant oocytes. Additionally, we also scored mitochondrial and ER volume to investigate if the volume of these organelles might be altered in the *Mater^tm/tm^* oocytes. We found that, while the volume fractions of mitochondria and ER were similar, the volume of lipid droplets increased approximately 7-fold in *Mater^tm/tm^* oocytes (Fig. 4C). The finding that mitochondrial volume fraction did not appear to be affected in *Mater^tm/tm^* oocytes is interesting in light of previous immuno-EM data showing that MATER localizes, in part, to mitochondria. Experiments are being carried out to address this observation. In support of our EM findings on the increase in lipid droplet volume fraction in *Mater^tm/tm^* oocytes, we also noticed that the morphology of these oocytes seems distinct from *Mater^+/+^* oocytes in that brown aggregates were observed in *Mater^tm/tm^* oocytes under the dissecting microscope. To test if these aggregates were LDs, we stained live *Mater^+/+^* and *Mater^tm/tm^* oocytes with Nile red and the merged images show that the aggregates are, in fact, LDs. (Fig. 4B). Finally, while not quantitated, we also noticed that the LDs also tended to form larger clusters in *Mater^tm/tm^* oocytes. Quantitation of the CPLs and organelles is shown in Figure 4C.

## Discussion

In this manuscript, we define a new role for MATER by showing that this maternal effect gene product appears to be required for CPL formation. Our EM morphometric analysis of the volume of CPLs in wild type and *Mater^tm/tm^* oocytes provides the most direct support of this conclusion with results showing that the volume fraction of CPLs is reduced by ∼10-fold in *Mater^tm/tm^* oocytes when compared to wild type oocytes. It is important to note here that the *Mater^tm/tm^* mice used in this study were derived from the same mice that were originally characterized as *Mater* null by Tong et al [8]. Subsequently, however, Ohsugi et al [28] later documented that these mice actually express low levels of MATER protein, and thus termed the mice *Mater^tm/tm^* or hypomorphs. We confirmed that these mice do express low levels of MATER protein and thus used the *Mater^tm/tm^* hypomorph terminology for this report. Interestingly, in our study, the observed reduction in CPL volume roughly correlates with the 90% reduction in MATER protein in the *Mater^tm/tm^* oocytes, suggesting that there may be a direct 1∶1 stoichiometry between MATER and CPLs. In other words, decreasing MATER protein concentrations would result in a corresponding decrease in lattice number. Thus, by extrapolation, we predict that lattices would be absent from true *Mater* null oocytes.

With respect to the role of MATER in lattice formation, while it is possible that MATER functions indirectly (as an upstream signaling factor for CPL formation, for example), we predict that MATER's role in lattice formation is direct via associations with other CPL components. This prediction is based on our observation that PADI6 (a *bona fide* CPL component) and MATER co-localize with each other throughout the cytoplasm and on Tashiro's immuno-EM finding that MATER localizes to the CPLs [30]. Therefore, MATER appears to represent a component of the CPL superstructure. Further, the observation that PADI6 solubility is increased in *Mater^tm/tm^* oocytes suggests that MATER plays a direct role in helping to nucleate CPL-associated proteins into the mature CPL superstructure and that, in the absence of MATER, CPL-associated proteins are rendered more soluble.

As noted in the introduction, MATER has also recently been found to be associated with, and required for, the formation of another supramolecular complex, termed the SCMC, which also contains FILIA, TLE6, and FLOPED. Further investigation into the molecular nature of this complex finds that *Mater*, *Filia*, and *Floped*-null mice are essential for development beyond the early cleavage stages of development. At the molecular level, FPLC and immunoprecipitation analysis suggests that the SCMC is formed by a direct interaction between FLOPED, MATER and TLE6, while FILIA appears to associate with the SCMC via direct interaction with MATER alone. At the subcellular level, these proteins appear to co-localize at the oocyte subcortex and this localization becomes asymmetrically restricted to apical cytocortex of two-cell embryos. In morulae, however, expression of the SCMC proteins is limited to the non-opposed cytocortex of outer blastomeres and these proteins are not observed within the inner cells. This restricted localization pattern has lead to the hypothesis that the SCMC structure may provide a molecular marker of embryonic cell lineages and possibly cell fate determinations [11]. While the role of the SCMC in early development remains to be elucidated, analysis of *Filia*-null mice suggests that this maternal factor plays an important role in integrating the spatiotemporal localization of regulators of euploidy and cell cycle progression during early development [12].

Interestingly, a very recent report by another group [30] has further characterized *Floped*-null mouse oocytes by electron microscopy and found that CPLs are also absent from these mutant oocytes, thus indicating that this SCMC protein is also required for lattice formation. Additionally, they found that, while confocal immunofluorescence (IF) analysis of isolated oocytes/embryos suggested that FLOPED localized to the cytocortex as shown previously [11], immuno-EM analysis indicated that FLOPED primarily localized at the CPLs throughout the cytoplasm. The investigators then predicted that this conflict in FLOPED subcellular localization patterns was due to the inability of anti-FLOPED antibodies to penetrate the cortex of isolated oocytes/embryos, thus resulting in a strong cortical FLOPED staining pattern. They then tested this prediction by staining paraffin embedded cross-sections of oocytes and embryos and found that, under these conditions, FLOPED staining was seen throughout the cytoplasm and was not concentrated at the cortex. Taken together, the findings by Tashiro et al. indicate that FLOPED primarily localizes to the CPLs and is also required for lattice formation.

In this report, we first document the co-localization of MATER and PADI6 at the cortex of non-extracted oocytes/embryos, and throughout the cytoplasm of Triton extracted oocytes. We predict that the punctate co-localization of PADI6 and MATER throughout the cytoplasm of Triton extracted oocytes is reflective of the localization of these maternal factors to the Triton-resistant CPLs. This prediction is supported by our previous immuno-EM finding showing that PADI6 primarily localizes to the CPLs and by the new Tashiro publication [30] which shows that, anti-MATER coated gold particles also localize to the CPLs. While we currently do not fully understand why PADI6 and MATER confocal IF staining is primarily limited to the cortex in non-extracted oocytes, the new findings on FLOPED localization by Tashiro raise the possibility that the observed cortical localization of PADI6 and MATER is artifactual in nature. In fact, we have found that the ratio of cortical versus cytoplasmic PADI6 and MATER staining in intact oocytes can vary depending on the fixation and immunostaining conditions used [27], [28]. Alternatively, it is also possible that MATER and PADI6 are associated with the SCMC at the cortex and with the CPLs throughout the cytoplasm. The hypothesis that PADI6 is associated with the SCMC is supported by previous work showing that PADI6 potentially associates with FLOPED, a component of the SCMC [11]. If this hypothesis is correct, then it is possible that the SCMC and CPL complexes are structurally related and may exist as a continuum that cannot be resolved by electron microscopy. Thus, PADI6, MATER, and likely other proteins could be found to shuttle between the CPLs and the SCMC. Interestingly, our FPLC data may provide some support for this prediction. Our analysis shows that PADI6 and MATER appear to co-elute in three separate fractions. The initial high molecular weight fraction (∼670 to 2,000) is similar in mass to that shown for MATER and other SCMC components in the previous report [11]. This finding supports the hypothesis that PADI6 is a component of the SCMC. In our study, the observed midrange ∼200 to 670 kDa co-elution peak raises the possibility that MATER and PADI6 may also be associated with a smaller complex that either may not be directly associated with a specific structure. We note that the lowest molecular weight MATER and PADI6 co-elution peaks are well below the 200 kDa mass marker and predict these fractions represent monomeric forms of PADI6 and MATER. While it is currently unclear why Li et al did not also observe the midrange and lower MW peaks for MATER in their study, a comparison of protocols finds that we utilized different sized columns which may have affected protein resolution. Additionally, we also added 0.1% Trition to our oocyte lysis buffer, which may have affected protein solubility. It currently remains unclear whether the association between PADI6 and MATER is direct or indirect. For this study, we attempted numerous co-immunoprecipitation experiments and were not able to show an interaction between PADI6 and MATER. Interestingly, given that PADI6 was identified as a potential interacting partner with FLOPED and that FLOPED directly interacts with MATER, it is possible that PADI6 and MATER indirectly associate via interactions with FLOPED.

An unexpected finding from this study was that, in addition to the reduction of CPLs in *Mater^tm/tm^* oocytes, the volume of lipid droplets was significantly increased in the mutant oocytes. LDs are dynamic organelles that are primarily thought to store energy in the form of neutral lipids such as triacylglycerols and sterol esters [31]. In spite of the significant progress in LD research in recent years, the fundamental mechanisms by which LDs function remain mostly unknown. This is particularly true for mammalian oocytes, as there are only a few reports suggesting a role for LDs in oocyte function [32]–[35]. Interestingly, LD accumulation is frequently seen in pathologic conditions such as apoptosis, cancer, and inflammation [36]. A recent report has found an interaction between MATER and PKC {epsilon}, which is involved in the anti-apoptotic pathway [36], [37]. If MATER is indeed associated with an anti-apoptotic signaling pathway, LD accumulation in *Mater^tm/tm^* oocytes may be associated with apoptotic events. Importantly, our findings indicate that *Mater^tm/tm^* mice represent a valuable tool to study LD function in oocytes.

To conclude, in this study we show that MATER is required for CPL formation and also appears to represent a component of the CPL superstructure. In light of the new finding by Tashiro that another SCMC component, i.e. FLOPED, is also required for CPL formation, and that PADI6 was identified at a putative FLOPED interacting proteins, our new findings now highlight a potential relationship between the SCMC and the CPLs. Additionally, given the oocyte/embryo-restricted nature of PADI6 and MATER and that these factors are all essential for early cleavage divisions, these findings further highlight the importance of the CPLs in mediating the oocyte-to-embryo transition. Furthermore, given that MATER and PADI6 are both expressed in human oocytes, a better understanding of the role of murine MATER, PADI6, and the lattices in early developmental events may provide insight into human infertility.

## Materials and Methods

### Ethics statement

Animals were bred and maintained in accordance with Cornell animal care guidelines and animal protocol number 2007-0113 was approved by the Cornell Institutional Animal Care and Use Committee before implementation.

### Animals

The generation and validation of the *Mater^tm/tm^* mouse strain has been described previously [8]. CD-1 breeding mice were purchased from commercial vendors.

### Collection and culture of oocytes and embryos

Oocytes were harvested from 4- to 6-week-old *Mater^+/+^* and *Mater^tm/tm^* mice. GV-stage oocytes were harvested 46–48 hours after intraperitoneal injection of 5IU pregnant mare serum gonadotrophin (PMSG). 46–48 hours post PMSG, 5IU of human chorionic gonadotrophin (hCG) was administered intraperitonealy and Metaphase II (MII) oocytes were harvested 12–14 hours later. Oocytes were collected in M2 medium (Sigma) and 5 µM milrinone (Sigma) or 200 µM IBMX (Sigma) was added to the media when GV oocytes were used. For collection of preimplantation embryos, mice were superovulated with PMSG/hCG as above, mated, and two-cell and four-cell embryos were collected 2 and 3 days later, respectively.

### Confocal microscopy

Immunostaining and Triton extraction procedures for oocytes have been described elsewhere (Yurttas et al., 2008). Oocytes and embryos were fixed in 4% paraformaldedyde (PFA) (EM Sciences) for 30 minutes. For extraction, oocytes were incubated in extraction buffer containing 0.1M KCl, 20 mM MgCl_2_, 3 mM EGTA, 20 mM HEPES (pH 6.8), 0.1% Triton X-100 and 1× Complete Protease Inhibitor Cocktail (Roche) for 10 minutes and rinsed in PBS quickly and fixed. Oocytes were permeabilized with 0.5% Triton X-100 in PBS for 30 minutes, washed and incubated with rabbit anti-MATER (1∶1000) (Tong et al., 2004) or guinea pig anti-PADI6 (1∶1000) (Wright et al., 2003) in IF buffer (1% BSA, 0.5% Normal Goat Serum in PBS) for 1 hour followed by another 1 h incubation with the appropriate Alexa Fluor-conjugated (1∶450) secondary antibody (Molecular Probes) in IF buffer. Oocytes were mounted on slides with Slowfade Gold antifade agent (Molecular Probes) and imaged using an LSM 510 laser scanning confocal microscope (Carl Zeiss). Confocal microscope settings for comparison of MATER expression between *Mater^+/+^* and *Mater^tm/tm^* oocytes were identical. For Nile red staining, oocytes were quickly washed 5 times in M2 medium, 3 times in MEM alpha medium (Invitrogen), and then incubated with Nile red (Sigma, 5 µg/1 ml) in MEM alpha medium for 5 minutes. Oocytes were briefly washed in PBS/PVA and PBS, attached to MatTek dishes (MatTek Corporation), which were then filled with MEM alpha. All procedures were carried out at room temperature.

### Gel Filtration Chromatography

Eighty GV oocytes were collected from ovaries of CD-1 female mice. Using an AKTApurifier FPLC System (GE Healthcare), oocyte lysates were chromatographed on a superpose 6 K9/30 column pre-equilibrated with several column volumes of chromatography buffer containing 154 mM NaCl, 50 mM Na phosphate (PH 7.4), 5 mM EDTA, and 0.02% NaN3. Elution positions of molecular weight standards (Sigma) were used to calibrate the column and determine the void volume. Fractions (1.0 ml) were transferred to a PVDF membrane by slot blot manifold (Hoefer PR 648) and analyzed by immunoblot. FPLC experiments were repeated two times. The immunoblot was scanned and relative intensity of bands was measured by ImageJ.

### Western blotting

*Mater^+/+^* and *Mater^tm/tm^* GV oocytes (15 for non-extracted and 25 for extracted), were collected, lysed in 4X Laemmli buffer (8% SDS, 40% glycerol, 20% 2-mercaptoethanol, 0.25M Tris HCl pH 6.8, 0.008% bromophenol blue), and boiled for 10 minutes at 100°C. Samples were run on a 10% SDS-PAGE gel at 100–120 V and transferred to PVDF membranes at 250mA for 2 hours. Membranes were blocked with 5% milk in 0.1% TBS-T overnight at 4°C and subsequently incubated with PADI6 (1∶13,000), MATER (1∶12,000), and β-actin (1∶1,000, Abcam) antibodies for 1–2 hour followed by anti-guinea pig (1∶15,000), anti-rabbit (1∶15,000) HRP antibody (Jackson ImmunoResearch) for 1 hour. Blots were developed using Immobilon Western HRP Chemiluminescent Substrate (Millipore). Films were scanned and densitometry was performed by Adobe photoshop CS4 program.

### Transmission electron microscopy

Electron microscopy was performed as described elsewhere [24] with the following modifications. GV oocytes were collected from age-matched 4–6 week old *Mater^+/+^* and *Mater^tm/tm^* mice (three mice each) and immediately fixed with 2.5% Glutaraldehyde (EM Sciences), 4% PFA, 0.1% tannic acid and 0.01M MgCl_2_ in 0.1M sodium cacodylate buffer (pH 7.3) at room temperature for 2 hours then overnight at 4°C. The oocytes were post-fixed with 1% osmium teroxide in cacodylate buffer for 1 hour, en bloc stained with 2% uranyl acetate, dehydrated in a graded ethanol series and then embedded in LX-112 resin (Ladd Research). Thin sections (50–70 nm) were cut with a diamond knife (Diatome) on an AO/Reichert ultramicrotome and picked up on nickel 200 mesh thin bar grids. Grids are contrast stained with 2% uranyl acetate followed by Sato's modified lead stain. Samples were examined by a FEI T12 TWIN transmission electron microscopy (TEM) at 100kV and images were collected with a Gatan Orius® dual-scan CCD camera.

### Morphometric analysis

For morphometric analysis, TEM images (x 11,500) were overlayed with a 2106 cross point grid (0.8 cm spacing) using ImageJ, and points occupied by CPLs, LDs, mitochondria, and ER were scored using the point-counting method [29]. The scoring was performed on 18 randomly spaced thin sections, with 3 sections from each of 6 different oocytes, which were obtained from 3 independent biological replicates of both *Mater^+/+^* and *Mater^tm/tm^* mice.

### Statistical analysis

Means and standard error of the mean (SEM) were calculated for organelle volume fraction of *Mater^+/+^* and *Mater^tm/tm^* oocytes and statistical significance was calculated using a two-tailed *t*-test in Microsoft Excel 2007 program. P-value<0.05 was regarded as statistically significant. All experiments were repeated at least three times unless written otherwise.
